# Supplementary material for: Effect of Anti-Osteoporotic Treatments on Circulating and Bone MicroRNA Patterns in Osteopenic ZDF Rats
Source: Int J Mol Sci. 2022 Jun 10;23(12):6534. doi: 10.3390/ijms23126534 (PMC9224326; doi:10.3390/ijms23126534)
Supplement: Supplementary file 1 [file ijms-23-06534-s001.zip › ijms-1736367-supplementary.pdf]

Effect of anti-osteoporotic treatments on circulating and bone microRNA patterns in osteopenic ZDF rats

David Carro Vázquez[1], Lejla Emini[2], Martina Rauner[2], Christine Hofbauer[2], Johannes Grillari[3,4,5], Andreas B. Diendorfer[1], Richard Eastell[6], Lorenz C Hofbauer[2] and Matthias Hackl[1,5]

Supplementary Materials:

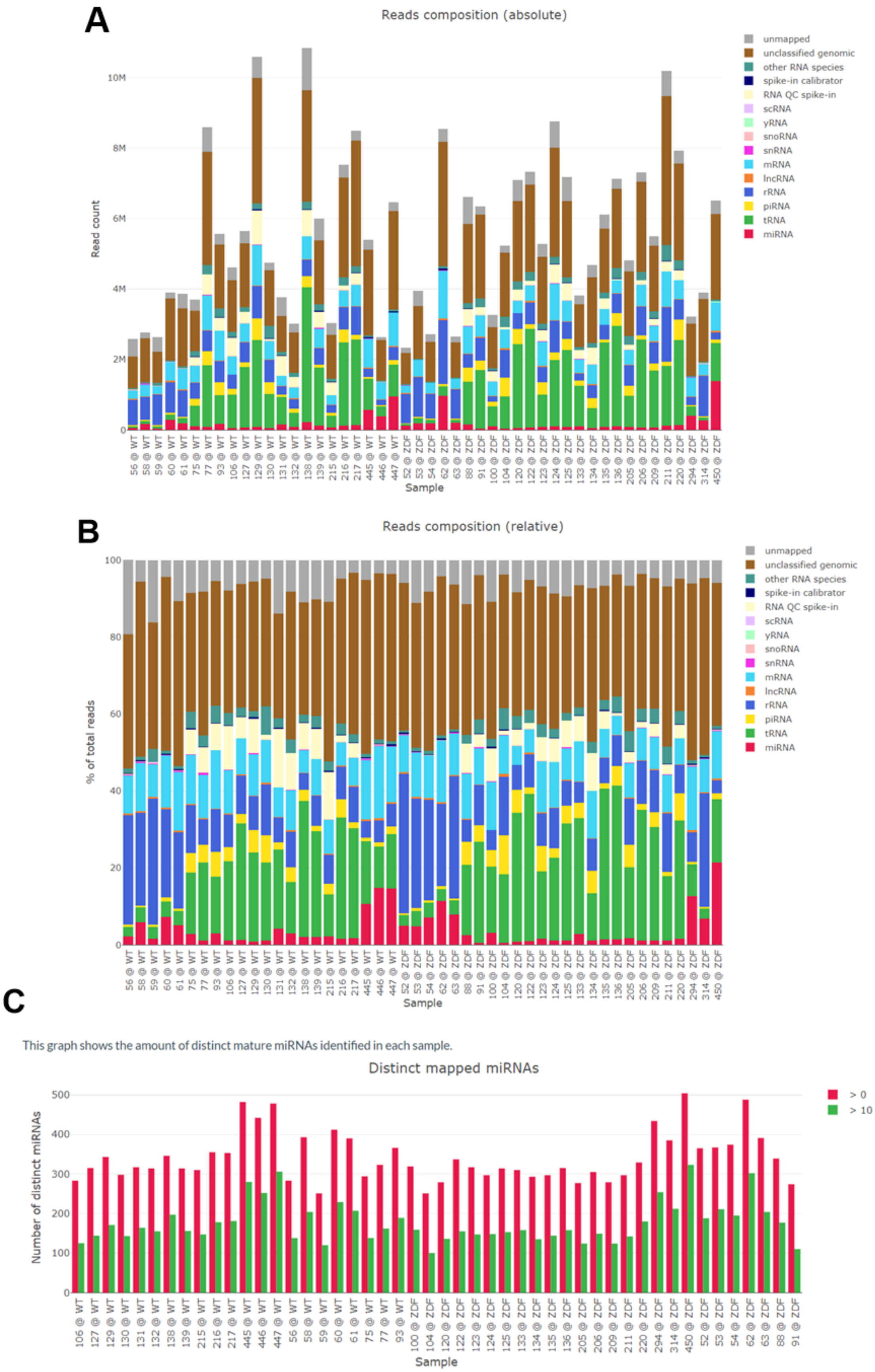

Supplementary Figure S1. NGS data quality control. A: Total reads obtained per sample. B: Relative reads mapping against miRNA reads. C: Amount of distinct mature miRNAs identified in each sample.

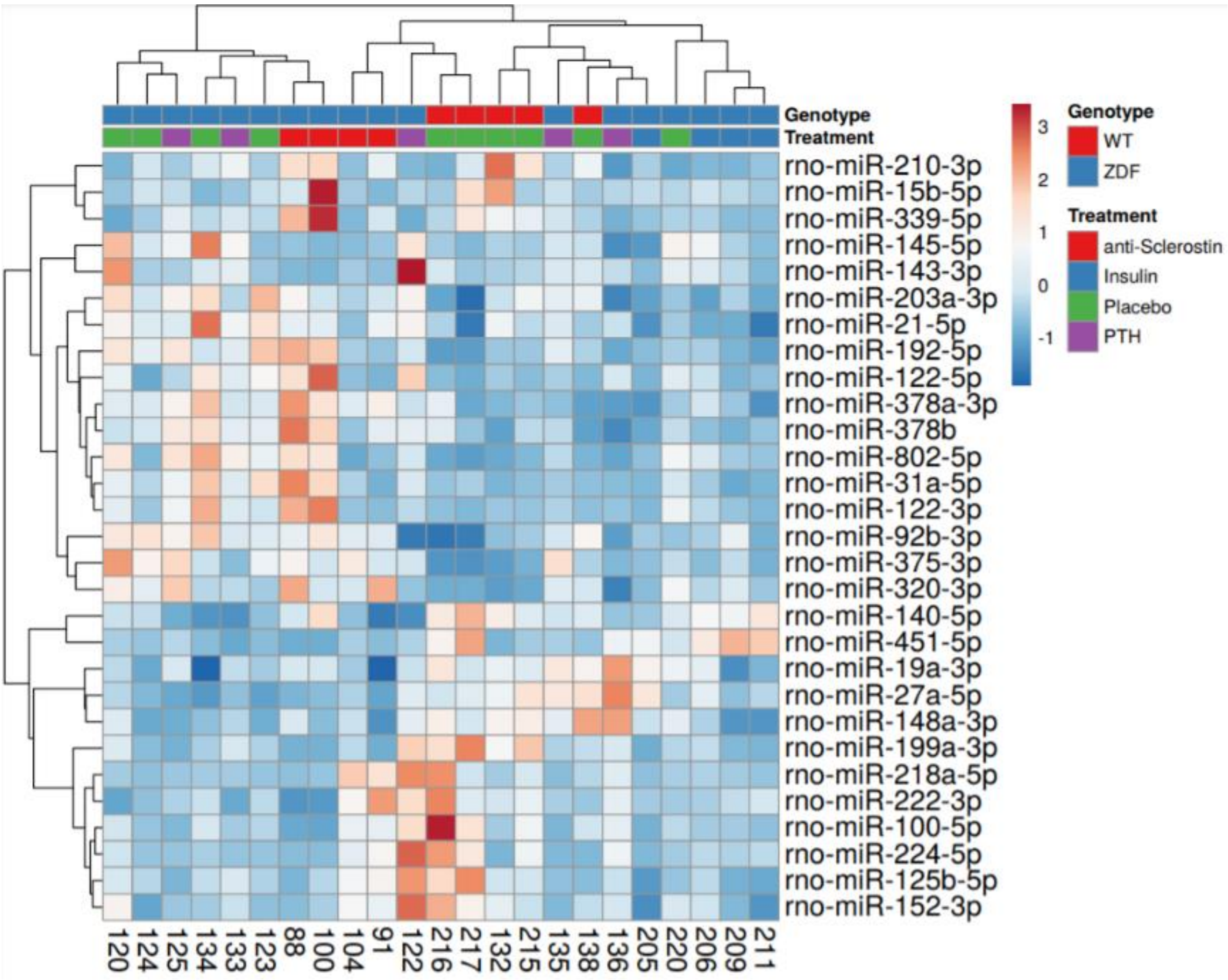

Supplementary Figure S2. Heatmap representations with top 30 miRNAs by significance level of dysregulation in serum (with p-value < 0.1). Placebo WT, n =5; Placebo ZDF, n=5, PTH ZDF, n=4-5; insulin ZDF, n=4; anti-scl ZDF, n=4. Heatmaps show groups of miRNAs merged by the smallest distance with the ClustVis online tool. Y-axis indicates the circulating miRNA detected by NGS; x-axis indicates the animal ID in which each circulating miRNA was detected.

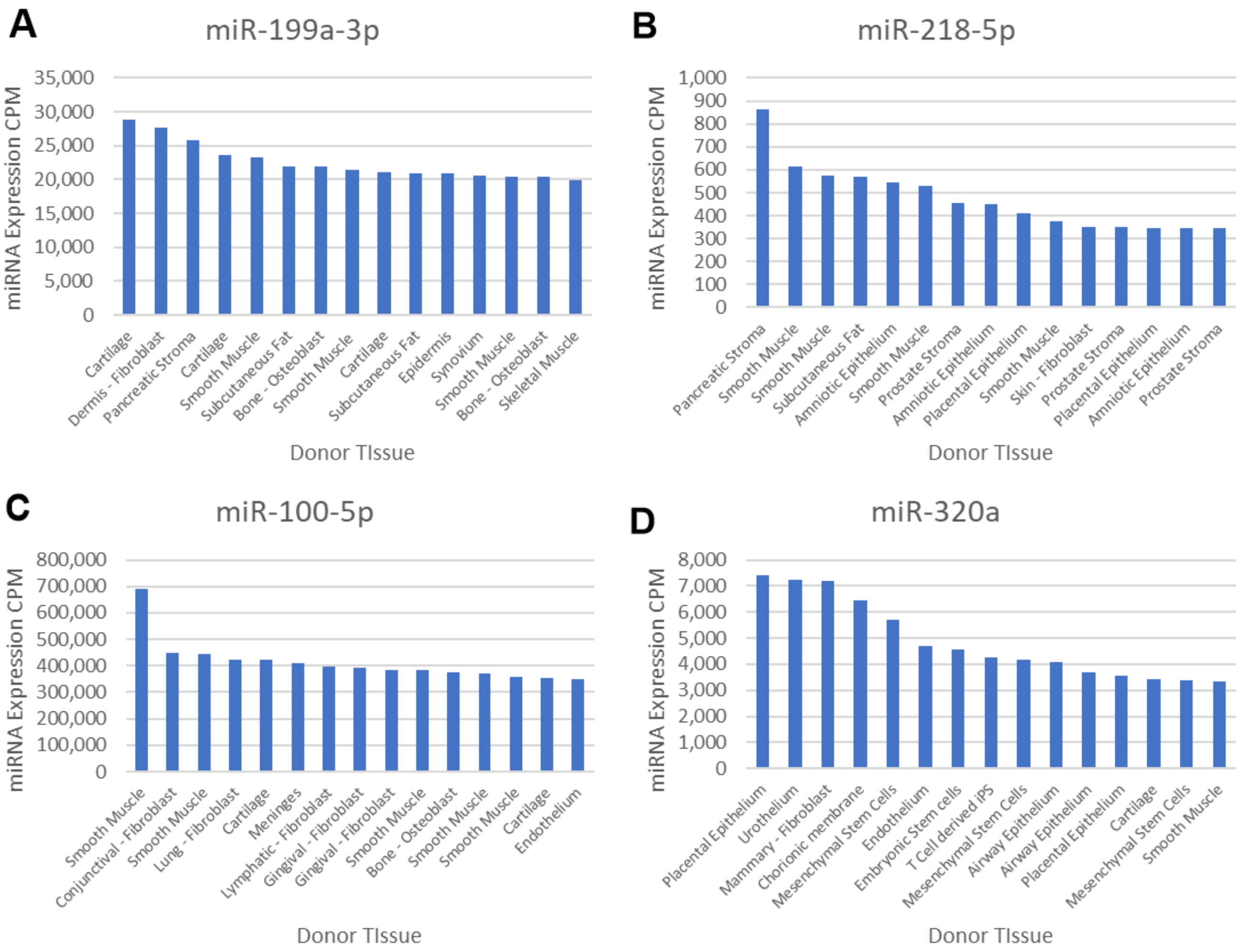

Supplementary Figure S3. Expression profile across cell and tissue types of the miRNAs not rescued by any treatment obtained by the FANTOM5 atlas browser. The four circulating miRNAs dysregulated in ZDF rats and not rescued by any of the treatments were searched in the FANTOM5 browser and their expression profiles across the top 15 cell and tissue types were obtained. Y-axis indicates the level of expression in CPM of the miRNA for each analyzed cell/tissue sample, x-axis shows the position of each cell/tissue sample where the miRNA was detected.

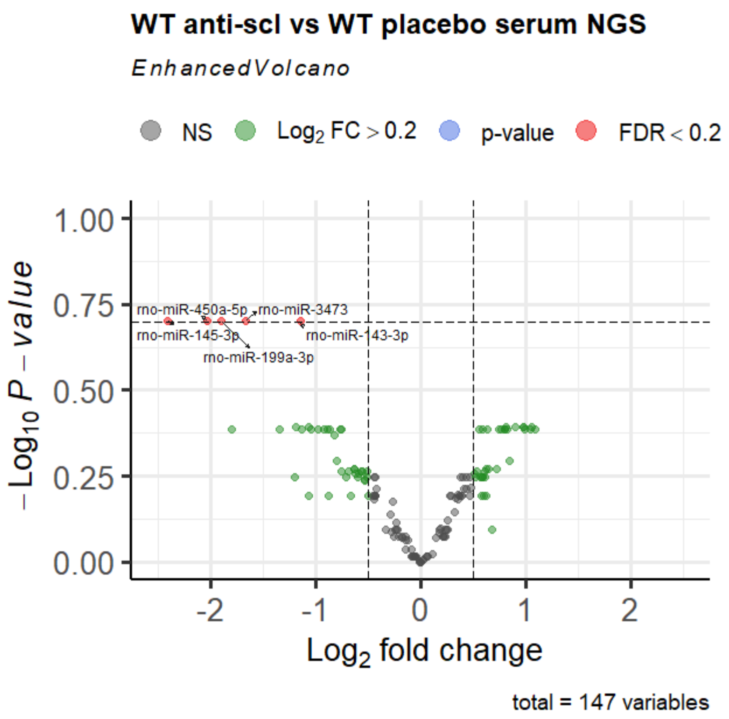

Supplementary Figure S4. NGS-discovery of miRNA changes in serum of WT rats under anti-sclerostin treatment. Volcano plot depicts the log2 transformed fold change and log10 transformed adjusted p-values for the contrast *(WT Placebo, n=5; WT anti-sclerostin, n=5)* in serum.

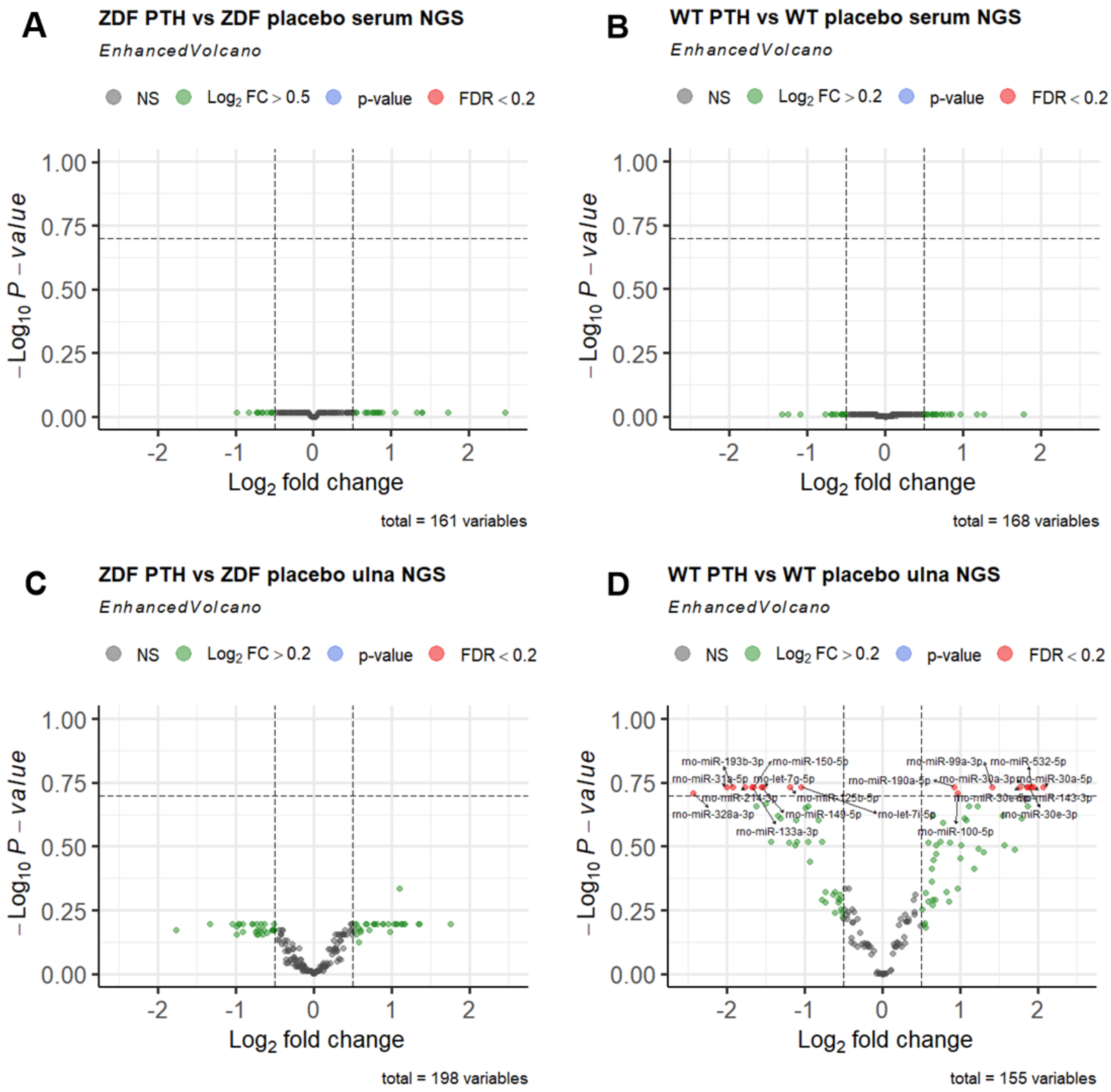

Supplementary Figure S5. NGS-discovery of miRNA changes in serum (A-B) and ulna bone tissue (C-D) of ZDF and WT rats under PTH treatment. A-B) Volcano plots depict the log2 transformed fold change and log10 transformed adjusted p-values for the different contrasts in serum of ZDF-Placebo rats and ZDF-PTH rats (A) and of WT-Placebo rats and WT-PTH rats (B). C-D) Volcano plots depict the log2 transformed fold change and log10 transformed adjusted p-values for the different contrasts in ulna bone of ZDF-Placebo rats and ZDF-PTH rats (C) and of WT-Placebo rats and WT-PTH rats (D). n=5 for all groups.

Supplementary Table S1. Pathway enrichment using the KEGG, Reactome and DisGeNET databases. Several pathways significantly enriched with an adjusted p value < 0.2 (FDR < 20%) were identified for the twelve miRNAs dysregulated in serum. The top targeted genes most commonly present in the identified pathways are highlighted (15 genes).

Database	Pathway	Hits	P-value	FDR
KEGG	Focal adhesion	**RHOA**; MET; ACTB; **AKT1**; IGF1R; **XIAP**; **ITGB1**; **VEGFA**; **FN1**; **RAC1**; LAMC1; **THBS1**	$1.5x10^{-6}$	$7.6x10^{-5}$
	ECM receptor interaction	CD47; **ITGB1**; CD44; **FN1**; LAMC1; **THBS1**	$3.3x10^{-4}$	0.0047
	Adherens junction	**RHOA**; MET; ACTB; IGF1R; **RAC1**	0.0011	0.011
	TGF-beta signaling	**RHOA**; SP1; BMPR2; **MYC**; **THBS1**	0.0024	0.018
	Axon guidance	**RHOA**; **ITGB1**; NRP1; **RAC1**; SRGAP1	0.01	0.06
	Cell cycle	**ATM**; CDK6; **MDM2**; **CDKN1A**; **MYC**	0.012	0.07
	mTOR Signalling	**AKT1**; **VEGFA**; **HIF1A**	0.014	0.073
	VEGF signaling	**AKT1**; **VEGFA**; **RAC1**	0.054	0.18
	Endocrine and other factor-regulated calcium reabsorption	ATP1B1; GNAS	0.062	0.19
Reactome	Signaling by Wnt	**RHOA**; SOX4; **TNRC6B**; **AKT1**; XIAP; USP34; KMT2D; **RAC1**; **TNRC6A**; **AGO3**; **MYC**; CLTC	$7.1x10^{-5}$	0.0018
	Cellular Senescence	SP1; **TNRC6B**; **ATM**; CDK6; **MDM2**; **TNRC6A**; **AGO3**; **CDKN1A**; IL6	$9.2x10^{-5}$	0.0018
	AKT phosphorylates targets in the cytosol	**AKT1**; **MDM2**; **CDKN1A**	$3.3x10^{-4}$	0.0034
	Signaling by SCF-KIT	**TNRC6B**; **AKT1**; **MDM2**; **RAC1**; **TNRC6A**; **AGO3**; **CDKN1A**	0.0017	0.0065
	Beta-catenin independent WNT signaling	**RHOA**; **TNRC6B**; **RAC1**; **TNRC6A**; **AGO3**; CLTC	0.0034	0.0097
	DAP12 signaling	**TNRC6B**; **AKT1**; **MDM2**; **RAC1**; **TNRC6A**; **AGO3**; **CDKN1A**	0.0038	0.098
	Syndecan interactions	**ITGB1**; **FN1**; **THBS1**	0.004	0.0098
	Signaling by NOTCH	**TNRC6B**; **TNRC6A**; **AGO3**; **HIF1A**; **MYC**	0.0051	0.011
DisGeNET	Complications of Diabetes Mellitus	**VEGFA**; **HIF1A**	$5x10^{-4}$	0.0085
	Abnormality of bone marrow cell morphology	**ATM**; **MYC**	$5x10^{-4}$	0.0085

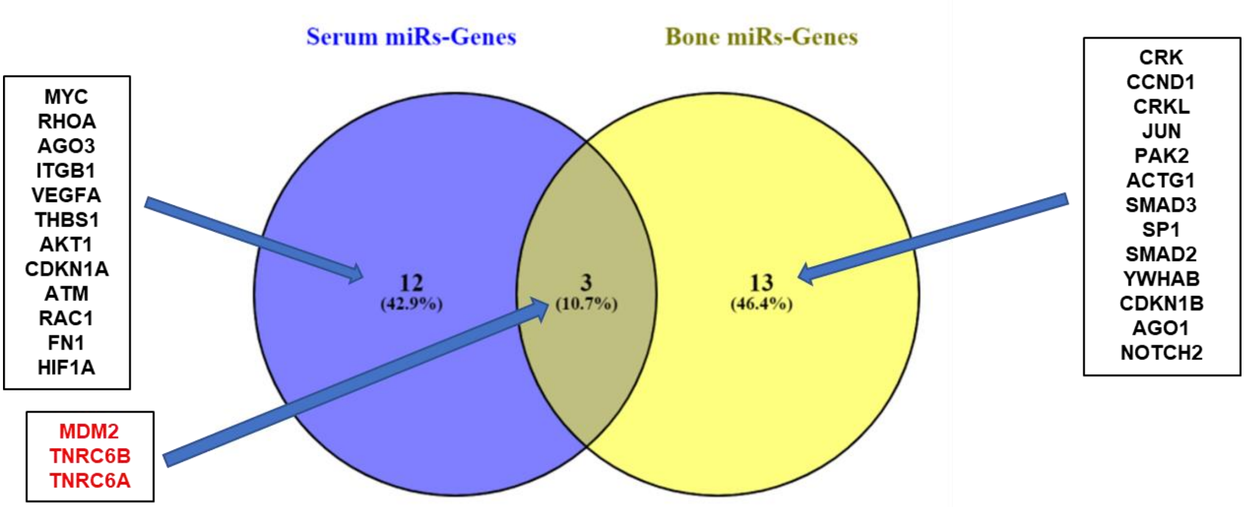

Supplementary Figure S6. Most relevant genes targeted by dysregulated miRNAs in serum (Supplementary Table S1) and in ulna bone tissue (Table 3) in ZDF rats compared to WT rats under placebo treatment. Comparison of the top targeted genes most commonly present in the identified pathways (Figure 5, Supplementary Figure 6) using miRNAs dysregulated in serum and in ulna bone (FDR threshold of 0.2). VENN diagram with the overlapping genes between the two compared lists corresponding to the most relevant genes targeted by dysregulated miRNAs in the ZDF model in serum (left circle) and ulna bone tissue (right circle). The genes MDM2, TNRC6B and TNRC6A that are present in both lists are highlighted in red.
